# Inhibition of the p53/hDM2 protein-protein interaction by cyclometallated iridium(III) compounds

**DOI:** 10.18632/oncotarget.7369

**Published:** 2016-02-13

**Authors:** Li-Juan Liu, Bingyong He, Jennifer A. Miles, Wanhe Wang, Zhifeng Mao, Weng Ian Che, Jin-Jian Lu, Xiu-Ping Chen, Andrew J. Wilson, Dik-Lung Ma, Chung-Hang Leung

**Affiliations:** ^1^ State Key Laboratory of Quality Research in Chinese Medicine, Institute of Chinese Medical Sciences, University of Macau, Macao, China; ^2^ Department of Chemistry, Hong Kong Baptist University, Hong Kong, China; ^3^ School of Chemistry, University of Leeds, Leeds LS29JT, UK; ^4^ Astbury Centre for Structural Molecular Biology, University of Leeds, Leeds LS29JT, UK

**Keywords:** metal-based inhibitor, protein-protein interaction, p53, hDM2

## Abstract

Inactivation of the p53 transcription factor by mutation or other mechanisms is a frequent event in tumorigenesis. One of the major endogenous negative regulators of p53 in humans is *h*DM2, a ubiquitin E3 ligase that binds to p53 causing proteasomal p53 degradation. In this work, a library of organometallic iridium(III) compounds were synthesized and evaluated for their ability to disrupt the p53/*h*DM2 protein-protein interaction. The novel cyclometallated iridium(III) compound 1 [Ir(eppy)_2_(dcphen)](PF_6_) (where eppy = 2-(4-ethylphenyl)pyridine and dcphen = 4, 7-dichloro-1, 10-phenanthroline) blocked the interaction of p53/*h*DM2 in human amelanotic melanoma cells. Finally, 1 exhibited anti-proliferative activity and induced apoptosis in cancer cell lines consistent with inhibition of the p53/*h*DM2 interaction. Compound 1 represents the first reported organometallic p53/*h*DM2 protein-protein interaction inhibitor.

## INTRODUCTION

The p53 transcription factor is involved in the regulation of cell proliferation and apoptosis, DNA repair, angiogenesis, and innate immunity [1]. Wild-type p53 (wt p53) functions as a tumor suppressor gene and promotes cell cycle arrest or apoptosis in cancer cells. However, inactivation of p53 by mutation or other mechanisms is a frequent event in tumorigenesis [2].

One of the major endogenous negative regulators of p53 in humans is the human double minute 2 protein (*h*DM2), which is a ubiquitin E3 ligase that binds to p53 [3]. p53 and *h*DM2 interact with each other to create an autoregulatory feedback loop that regulates both the activity of p53 and the gene expression of *h*DM2 [4]. In this feedback loop, p53 activation increases the gene expression level of *h*DM2. In turn, *h*DM2 binds to and conceals the N-terminal transactivation domain of p53, and facilitates the nuclear export of p53, leading to p53 degradation by the ubiquitin proteasome pathway. Due to the role of *h*DM2 in negatively regulating the function of the p53 tumor suppressor protein, the overexpression of *h*DM2 has been detected in many types of cancer [5]. Many peptide-based or small-molecule disrupters of the p53/*h*DM2 interaction have been developed through inhibiting a well-defined hydrophobic surface pocket in *h*DM2 and three key hydrophobic residues of p53 projected from the same face of an α-helix [6]. To date, at least three small-molecule p53/*h*DM2 disrupters (RO5045337, RO5503781 and MI-888) have been advanced into clinical trials [7–9], however, none has yet been approved for clinical use.

The exploration of organometallic compounds containing transition metals as probes for chemical biology or as lead scaffolds for the design of potent inhibitors of pharmacologically-important molecular targets has received increasing attention in recent years [10–23]. Of particular interest are organometallic iridium compounds due to their tunable chemical and biological reactivities, as well as the distinctive kinetic inertness of the iridium(III) metal center. Another feature of organometallic iridium(III) compounds is that they are always six-coordinate with octahedral geometry [24–28]. Meggers and co-workers have pioneered the development of kinetically-inert organometallic iridium(III) compounds as potent and specific inhibitors of enzyme activity [29]. Moreover, Sadler and co-workers have conducted extensive investigations into organometallic iridium “half-sandwich” compounds as potent anticancer agents [30]. Sheldrick and co-workers have also studied a wide range of iridium(III) polypyridyl compounds that show promising selectivity for cancer cells over normal cells [31]. Meanwhile, our group has previously reported a cyclometallated iridium(III) compound that inhibited the trimerization of tumor necrosis factor alpha (TNF-α) [32], which represented the first example of an organometallic octahedral compound that inhibits PPIs. These studies highlight the increasing interest and importance given to the development of organometallic iridium(III) compounds as potential medicines. However, no metal-based inhibitor of the p53/*h*DM2 interaction has yet been reported in the literature. In this work, we set out to identify novel p53/*h*DM2 PPI disrupters *via* the screening of an in-house collection of kinetically-inert iridium(III) compounds. These efforts culminated in the identification of a novel cyclometallated iridium(III) compound **1** as the first metal-based disrupter of the p53/*h*DM2 PPI.

## RESULTS

### Synthesis and characterization of organometallic iridium(III) compounds

**Figure 1:**
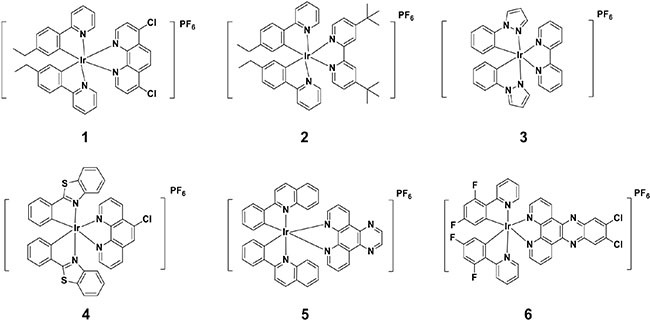
Chemical structures of kinetically-inert organometallic iridium(III) compounds 1–6

A library of structurally diverse, kinetically-inert organometallic iridium(III) compounds **1**–**6** (Figure 1) with a general structure [Ir(C^N)_2_(N^N)]^+^ were designed and synthesized. Among the structural motifs present in compounds **1**–**6** are aromatic and alkyl motifs, which could potentially form hydrophobic interactions with specific residues of the protein-protein interface, and halogen atoms (chlorine and fluorine), which are key groups in many approved drugs. Compounds **1**, **2** and **6** bear 2-phenylpyridine (ppy) C^N ligands substituted with ethyl or fluoro groups, while compound **3** bears 1-phenyl-*1H*-pyrazole C^N ligands. Compound **5** possesses 2-phenylquinoline C^N ligands, while compound **4** bears 2-phenylbenzothiazole ligands. With regards to the N^N ligand, compound **3** carries the 2, 2′-bipyridine (bpy) moiety, while compound **2** possesses a bpy ligand substituted with tert-butyl groups at the 4 and 4′ positions. Compounds **1** and **4** bear 1, 10-phenanthroline (phen) N^N ligands substituted with one or two chlorine atoms at different positions. Finally, compounds **5** and **6** carry larger N^N ligands with additional fused aromatic rings.

**Scheme 1:**
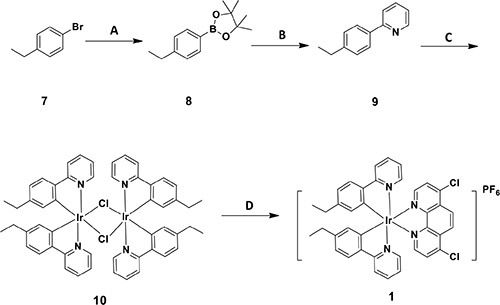
Synthetic pathway of cyclometallated iridium(III) compound 1 Reagents and conditions: **(A)** Bis(pinacolato)diboron, Pd(dppf)Cl_2_, AcONa, toluene, 100°C, N_2_; **(B)** 2-bromopyridine, Pd(PPh_3_)_4_, K_2_CO_3_, EtOH, reflux, N_2_; **(C)** IrCl_3_·3H_2_O, methoxyethanol/H_2_O = 3:1, 150°C; **(D)** 4, 7-dichloro-1, 10-phenanthroline, DCM/MeOH = 1:1, reflux, NH_4_PF_6_, H_2_O, Et_2_O.

The cyclometallated iridium(III) compound **1** [Ir(eppy)_2_(dcphen)](PF_6_) (where eppy = 2-(4-ethylphenyl)pyridine and dcphen = 4, 7-dichloro-1, 10-phenanthroline) was prepared as shown in Scheme 1. 1-Bromo-4-ethylbenzene **7** was converted to aryl boronate **8**, which was subsequently coupled to 2-bromopyridine *via* the Suzuki-Miyaura reaction to give C^N ligand eppy **9**. The reaction of **9** with IrCl_3_·3H_2_O gave the diiridium compound [(C^N)_2_Ir(μ-Cl)]_2_ **10**. Finally, dimer **10** was reacted with dcphen to generate **1**, which was isolated as the hexafluorophosphate salt by the addition of NH_4_PF_6_. The crude product was purified by column chromatography on silica to give **1** as a stable orange solid. The stability of **1** was investigated by UV-Vis and ^1^H NMR experiments (Supplementary Figures S1 and S2), which showed that **1** was stable in 90% [*d*_6_] DMSO/10% D_2_O or 80% acetonitrile/20% Tris-HCl buffer for at least 7 days. The synthesis of the other iridium(III) compounds and ligands are described in the Electronic Supplementary Information. All of the compounds in this work were characterized by ^1^H-NMR, ^13^C-NMR, high resolution mass spectrometry (HRMS) and elemental analysis.

### Compound 1 disrupts the p53/*h*DM2 interaction

**Figure 2:**
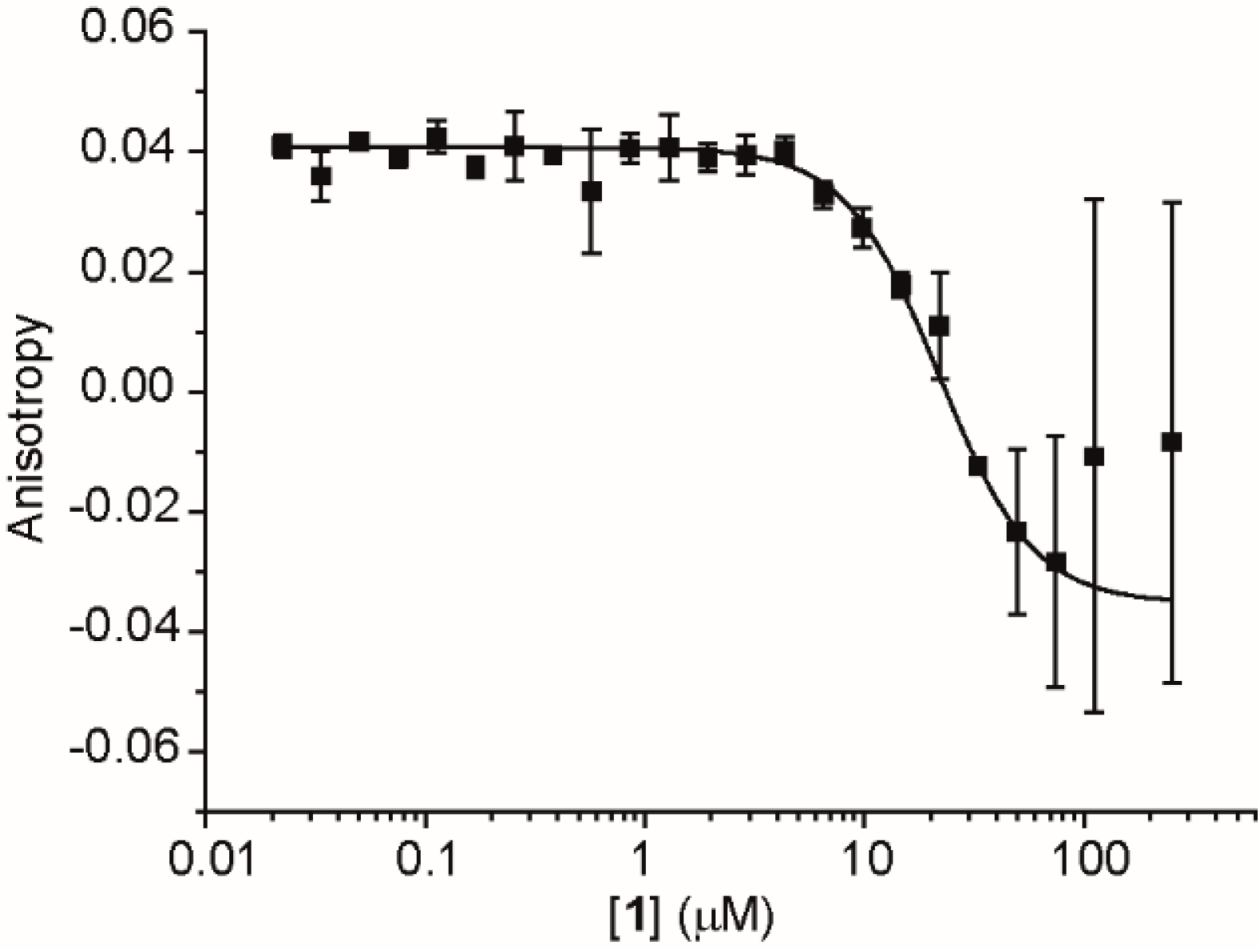
FA titration data of 1 **1** was incubated with 150 nM *h*DM2_17–126_ recombinant fragment and 50 nM fluorescein-labelled p53 peptide (p53_15–31Flu_), and fluorescence anisotropy was measured at 480ex/535em.

To experimentally test the library of the organo-metallic iridium(III) compounds as p53/*h*DM2 PPI inhibitors, a fluorescence anisotropy (FA) assay was performed as previously described [33]. *h*DM2_17–126_ (recombinant fragment) was incubated with fluorescein-labelled p53 peptide (p53_15–31Flu_) and serial dilutions of the organometallic iridium(III) compounds. The results revealed that the fluorescence anisotropy of the system was decreased significantly upon the addition of **1**, **5** and **6** (Figure 2 and Supplementary Figure S3, note: higher errors towards the lower asymptote which we ascribe to the absorbance of the iridium organometallic core). In contrast, the p53/*h*DM2 interaction was not significantly perturbed by the addition of **2**–**4**. The IC_50_ values of the active cyclometallated compounds against the p53/*h*DM2 interaction are presented in Table 1. Compound **1** was subsequently selected for further study as a potential disrupter of the p53/*h*DM2 interaction.

**Table 1:** IC_50_ values of the cyclometallated iridium(III) compounds 1–6 against the p53/hDM2 interaction as determined by a FA assay

Cyclometallated iridium(III) compounds	IC_50_/μM
**1**	16 (± 5)
**2**	No noticeable binding
**3**	No noticeable binding
**4**	No noticeable binding
**5**	21 (± 5)
**6**	33 (± 3)

**Figure 3:**
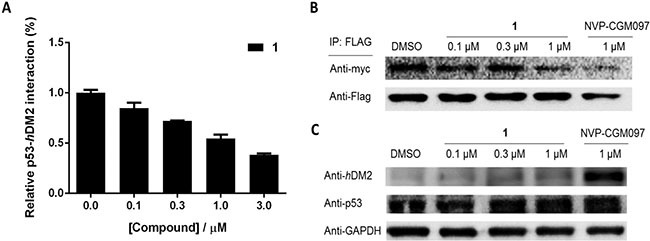
1 inhibits the interaction of p53/*h*DM2 in A375 cells without affecting protein expression levels **(A) 1** inhibits the p53/*h*DM2 NanoBRET protein-protein interaction. **(B) 1** inhibits the interaction of p53/*h*DM2 in A375 cells. The complex of p53/*h*DM2 in A375 cells was pulled down using anti-Flag magnetic beads and determined by probing with anti-Flag and anti-myc antibodies. **(C) 1** has no significant effect on the protein expression levels of p53 and *h*DM2 in A375 cells. The protein expression levels of p53 and *h*DM2 determined by probing with anti-p53 and anti-*h*DM2 antibodies.

We next employed the NanoBRET platform [34] to elucidate the effect of **1** on the p53/*h*DM2 PPI in live cells. Human amelanotic melanoma A375 cells were co-transfected with plasmids expressing p53-HaloTag and NanoLuc-*h*DM2. Transfected cells were then treated with vehicle, positive control compound NVP-CGM097 and **1** at various concentrations. The results revealed that **1** induced a dose-response decrease of bioluminescence resonance energy transfer (BRET) in treated cells, with an IC_50_ value of *ca.* 1 μM (Figure 3A). This result indicates that **1** was able to disrupt the p53/*h*DM2 PPI in A375 cells.

A pull-down assay was conducted to further examine the effect of **1** on the p53/*h*DM2 interaction. A375 cells were co-transfected with cDNAs encoding p53-Flag and *h*DM2-myc fusion proteins. After 6 h of treatment with NVP-CGM097 or **1**, p53/*h*DM2 complexes were immunoprecipitated using anti-Flag magnetic beads and analysed by immunoblotting. In the absence of **1**, *h*DM2 was precipitated together with p53. However, the amount of precipitated *h*DM2 was decreased with increasing concentrations of **1** or with NVP-CGM097 (Figure 3B). This result further confirms that **1** antagonized the interaction of p53/*h*DM2 in cells.

To assess whether **1** suppressed p53 or *h*DM2 protein expression levels, resulting in a decrease in the levels of precipitated protein detected in the pull-down assay, an immunoblotting experiment was performed. Immunoblotting analysis showed that **1** had no effect on the *h*DM2 and p53 protein expression levels, even at the highest concentration of 1 μM (Figure 3C). On the other hand, NVP-CGM097 significantly increased the expression of *h*DM2 protein at 1 μM, consistent with previous reports [35]. We also investigated the possible impact of the isolated ligands of **1** on the p53/*h*DM2 interaction and protein expression levels. Neither eppy nor dcphen ligands were found to have a significant effect on the p53/*h*DM2 interaction or protein expression levels (Supplementary Figure S4), suggesting that the inhibition of the p53/*h*DM2 interaction requires the assembly of the ligands into an intact complex. Taken together, these findings indicate that **1** significantly inhibited the p53/*h*DM2 interaction in A375 cells without affecting their protein expression.

### Compound 1 reactivates p53 transcriptional transactivation and induced apoptosis in cells

**Figure 4:**
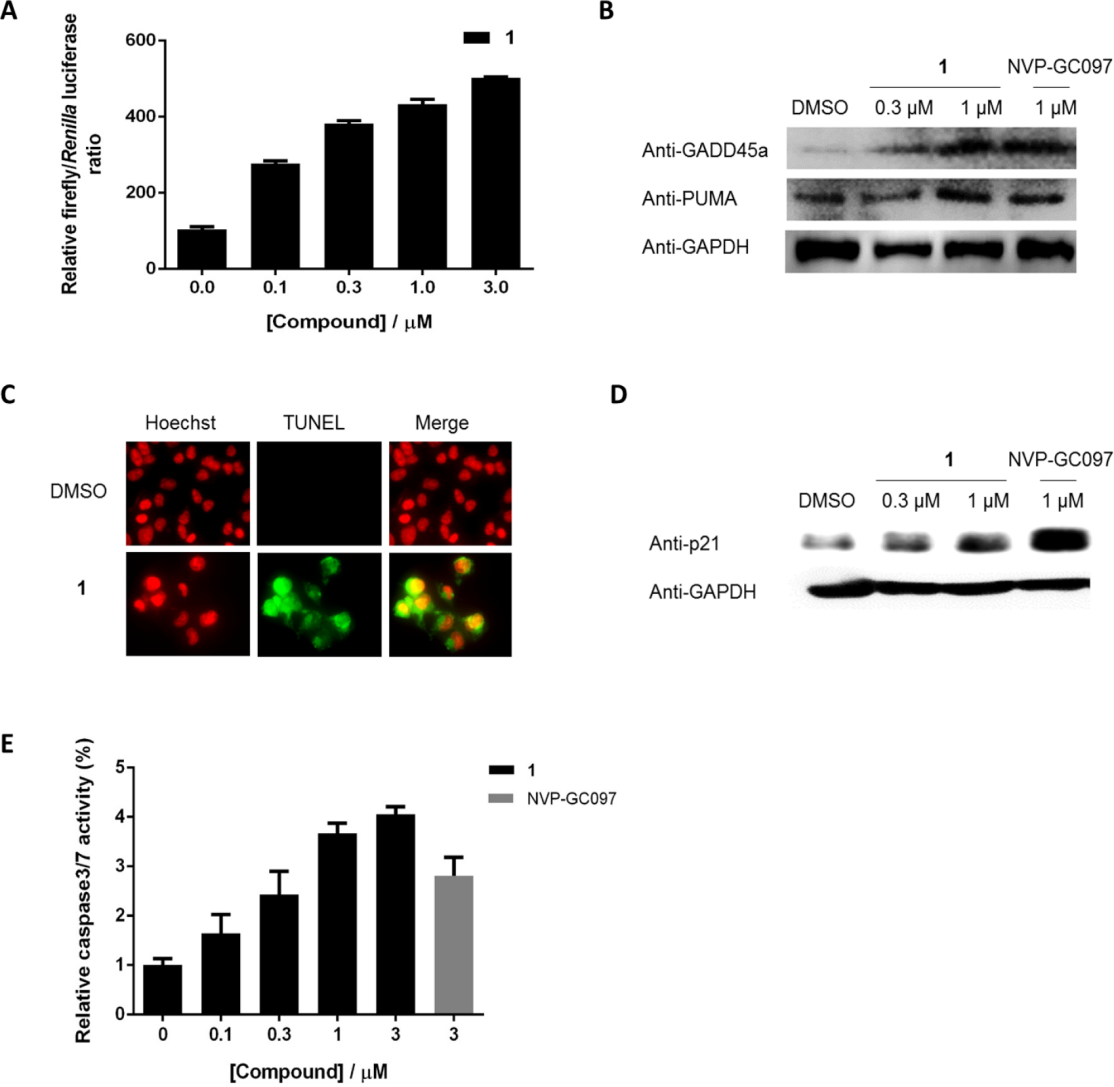
1 reactivates p53 transcriptional transactivation and induced apoptosis in cells **1** induces **(A)** p53-driven luciferase activity, **(B)** the expression of endogenous p53 targets GADD45α and PUMA, **(C)** DNA fragmentation, **(D)** p21 expression, and **(E)** caspase-3/7 activities in A375 cells.

We next studied whether **1** could reactivate the transcriptional transactivation function of p53 *via* blocking the p53/*h*DM2 interaction. Treatment of A375 cells carrying endogenous wt p53 and a p53-dependent luciferase reporter gene by **1** resulted in an increase in luciferase activity, indicating the activation of p53-direted transcription by **1** (Figure 4A). Furthermore, the expression of p53 target gene products GADD45α and PUMA were significantly increased by **1** in A375 cells (Figure 4B). These results indicate that the activation of GADD45α and PUMA gene expression in A375 cells by **1** is likely driven by its effects on wt p53.

Transactivation of p53 restricts cellular proliferation by inducing apoptosis and cycle arrest [36]. Therefore, a terminal deoxynucleotidyl transferase (TdT)-mediated dUTP nick and labelling (TUNEL) and Hoechst staining assay were then performed to investigate DNA fragmentation, a hallmark of apoptosis, in apoptotic cells. Notably, **1** was found to induce DNA fragmentation at a concentration of 0.3 μM (Figure 4C). Furthermore, **1** induced the expression of p21 (Figure 4D), a cyclin-dependent kinase (CDK) that is transcriptionally activated by p53 in response to DNA damage and acts to induce cell cycle arrest [37]. Finally, the effect of **1** on caspase-3/7, an apoptotic executioner under the control of p53, was investigated in A375 cells using a homogeneous luminescence assay. As shown in Figure 4E, **1** was able to significantly activate caspase-3/7 activities in a dose-dependent manner. Therefore, we reason that the induction of apoptosis by **1** in A375 cells is driven, at least in part, by the activation of p53 resulting from the disruption of the p53/*h*DM2 interaction.

### Compound 1 was toxic to cancer cells

Clinical observations have linked the overexpression of *h*DM2 with human cancers possessing wild-type p53 status [38, 39]. The cytotoxicity of **1** towards ten cancers cell lines with different p53 statuses (wt, mut or null) was subsequently examined assessed using the MTT assay (Supplementary Table S2). The results showed that **1** inhibited the growth of cancer cells at low micromolar concentration. Notably, **1** exhibited a greater toxicity towards cancer cell lines carrying wt p53 than those carrying mut p53, indicating that toxicity of **1** was wt p53-dependent. Therefore, we hypothesize that the ability of 1 to inhibit cancer cell proliferation may be due, at least in part, to its inhibition of p53/*h*DM2 interaction, leading to the activation of p53 transcriptional transactivation resulting in cell apoptosis.

## DISCUSSION

In this work, we have identified a novel kinetically-inert organometallic iridium(III) compound **1** as the first metal-based p53/*h*DM2 PPI disrupter. Compound **1** bearing 2-(4-ethylphenyl)pyridine (eppy) and 4, 7-dichloro-1, 10-phenanthroline (dcphen) ligands exhibited the most potent ability to disrupt the interaction between p53 and *h*DM2 among the six tested iridium(III) compounds, as revealed by a fluorescence anisotropy assay. Moreover, **1** was able to inhibit the interaction of p53/*h*DM2 cells without affecting their protein expression levels in A375 cells as determined by NanoBRET and pull-down experiments. **1** reactivated p53 transcriptional transactivation *in cellulo*, and also induced apoptosis and suppressed the growth of cancer cells, which we attribute, at least in part, to the disruption of the p53/*h*DM2 interaction by **1**.

Based on the FA results, a brief structure-activity relationship analysis of the six tested organometallic iridium(III) compounds can be performed. Comparison of compounds **1** and **2** indicates that the dcphen N^N ligand of **1** is superior for p53/*h*DM2 inhibitory activity compared to the bulky tert-butyl-substituted bpy ligand of **2**. Moreover, **3** and **4** had no noticeable effect on the p53/*h*DM2 interaction, indicating that their specific combinations of N^N and C^N ligands were undesirable for biological activity. Finally, **5** and **6** carrying larger N^N ligands with additional fused aromatic rings also showed moderate p53/*h*DM2 inhibitory activity, but were less potent than **1**. This suggests that the binding site of the target protein may be tolerant to presence of the additional planar aromatic rings of **5** and **6**, potentially *via* stacking interactions with aromatic residues, but not to the tert-butyl substituents of **2**, which are more sterically demanding in three dimensions. Taken together, these results suggest that size, electronic properties and steric properties of the organometallic compounds are important in determining their activity against the p53/*h*DM2 interaction.

The activation of p53 protein is a potential strategy in anti-cancer therapy. High p53 protein levels lead to apoptosis, while moderate p53 protein levels result in cell cycle arrest [40]. In humans, a major negative regulator of p53 is the E3 ubiquitin protein ligase *h*DM2/*m*DM2 pathway, the activation of which triggers the degradation of p53 [41]. To date, numerous small-molecule inhibitors of p53/*m*DM2 interaction have been discovered [40]. The first small-molecule inhibitor of p53/*m*DM2 interaction, 4, 5-dihydroimidazoline (nutlin, Roche), was demonstrated to bind to *m*DM2 and induce p53-dependent cell cycle arrest at low micromolar levels *in vitro* [42]. Several nutlin analogues (nutlin-2 and nutlin-3) and other structural classes of inhibitors, such as spiro-oxindoles and benzodiazepinediones, have demonstrated promising tumor growth inhibition and tumor shrinkage in animal models [40, 43]. *m*DM2 inhibitors derived from natural products, such as the prenylated xanthones α-mangostin (from the fruit of *Garcinia mangostana L.*) and gambogic acid (from the resin of *Garcinia hanburyi*), have also been identified, and were shown to bind in a similar manner to the nutlins [44]. In addition, peptide and stapled peptide inhibitors of the p53/*m*DM2 interaction have been shown to penetrate cells, bind tightly to *m*DM2 and induce p53 pathway activation with *in vitro* IC_50_ values ranging from 0.005 to 700 μM [45, 46]. However, the most potent peptide inhibitors exhibited only low cellular activities due to their poor cell permeability.

In comparison to known p53/*m*DM2 protein-protein interaction inhibitors, the iridium(III) compounds developed in this work possess similar abilities to activate p53 signaling and inhibit cancer cell growth *in vitro*, albeit with reduced potency. Nevertheless, given the status of these compounds as the first metal-based inhibitors of the p53/*h*DM2 interaction, we consider that compound **1** could be utilized as a structural starting point for the development of more potent anti-cancer compounds. Notably, the simple structure of compound **1** coupled with the modular nature of inorganic synthesis could feasibly allow many iridium(III) compounds to be synthesized and tested, in contrast to the relatively complex small molecule or peptide-based inhibitors of the p53/*m*DM2 interaction existing in the literature.

## MATERIALS AND METHODS

### General experimental

Chemicals were purchased from Sigma-Aldrich, J & K Scientific, Armar and TCI, and used as received; all solvents used were reagent grade. TLC was performed on Sorbent Technologies aluminum-backed Silica G TLC plates, and column chromatography was performed on silica gel 60 (Merck, 230–400 mesh). High resolution of mass spectrometry was carried out in the Department of Chemistry, Hong Kong Baptist University. ^1^H and ^13^C NMR were recorded on a 400 MHz (^1^H) and 100 MHz (^13^C) Bruker instrument using acetone-*d*_6_ or DMSO-*d*_6_ as the solvent. ^1^H and ^13^C chemical shifts are expressed in ppm relative to solvent peak (acetone-*d*_6_: ^1^H δ 2.05,^13^C δ 206.68, 29.92; DMSO-*d*_6_: ^1^H, 2.50, ^13^C, 39.5). The following abbreviations are used for signal patterns: s, singlet; d, doublet; dd, doublet of doublets; t, triplet; q, quartet; m, multiplet. All NMR data was acquired and processed using standard Bruker software (Topspin). Elemental analysis of the organometallic compounds was performed in Atlantic Microlab, Inc., USA. The synthetic procedures and photophysical properties of the iridium(III) compounds 1–6 used in this study are provided in the Supporting Information.

### Characterization of organometallic iridium(III) compounds

**1: [Ir(eppy)2(dcphen)](PF6)** (where eppy = 2-(4-ethylphenyl)pyridine and dcphen = 4, 7-dichloro-1, 10-phenanthroline), 4, 7-dichloro-1, 10-phenanthrolinebis(2-(4-ethylphenyl)pyridine)iridium(III) hexafluorophosphate Yield: 68.7%. ^1^H NMR (400 MHz, Acetone-*d*_6_) δ 8.71 (d, *J* = 6.4 Hz, 2H), 8.43–8.41 (m, 2H), 8.25 (d, *J* = 5.6 Hz, 2H), 8.17 (d, *J* = 8.0 Hz, 2H), 7.90–7.83 (m, 4H), 7.76 (d, *J* = 5.2 Hz, 2H), 6.95–6.91 (m, 4H), 6.28 (d, *J* = 1.2 Hz, 2H), 2.42 (d, *J* = 7.8 Hz, 4H), 1.03 (t, *J* = 7.8 Hz, 6H); ^13^C NMR (100 MHz, Acetone-*d*_6_) 168.7, 152.7, 150.5, 148.9, 147.4, 145.7, 142.8, 139.4, 131.9, 131.0, 128.7, 126.3, 125.8, 123.8, 123.4, 120.4, 29.8, 15.5; MALDI-TOF-HRMS: Calcd. for C_38_H_30_Cl_2_IrN_4_[M–PF_6_]^+^ : 805.1477, found: 805.5739; Anal.: (C_38_H_30_Cl_2_IrN_4_PF_6_+2H_2_O) C, H, N: calcd. 46.25, 3.47, 5.68; found. 46.04, 3.11, 5.67.

**2: [Ir(eppy)2(dtbpy)](PF6)** (where eppy = 2-(4-ethylphenyl)pyridine and dtbpy = 4, 4′-di-tert-butyl-2, 2′-bipyridine), 4, 4′-di-tert-butyl-2, 2′-bipyridinebis(2-(4-ethylphenyl)pyridine)iridium(III) hexafluorophosphate Yield: 70.3%. ^1^H NMR (400 MHz, Acetone-*d*_6_) δ 8.86 (d, *J* = 0.8 Hz, 2H), 8.17 (d, *J* = 8.0 Hz, 2H), 7.97 (d, *J* = 6.0 Hz, 2H), 7.96–7.90 (m, 2H), 7.79 (d, *J* = 8.0 Hz, 2H), 7.78–7.75 (m, 2H), 7.70 (d, *J* = 2.0 Hz, 2H), 7.10–7.07 (m, 2H), 6.90 (d, *J* = 2.0 Hz, 2H), 6.21 (d, *J* = 8.0 Hz, 2H), 2.40 (d, *J* = 7.6 Hz, 4H), 1.41 (s, 18H), 1.01 (t, *J* = 7.6 Hz, 6H); ^13^C NMR (100 MHz, Acetone-*d*_6_) 169.0, 164.8, 156.8, 152.3, 151.0, 149.8, 147.4, 142.6, 139.3, 131.7, 126.4, 125.8, 123.8, 123.0, 122.8, 120.4, 36.4, 30.2, 29.8, 15.5; MALDI-TOF-HRMS: Calcd. for C_44_H_48_IrN_4_[M–PF_6_]^+^: 825.3508, found: 825.1686; Anal.: (C_44_H_48_IrN_4_PF_6_+H_2_O) C, H, N: calcd. 53.48, 5.10, 5.67; found. 53.46, 4.86, 5.78.

**3: [Ir(ppz)2(bpy)](PF6)** (where ppz = 1-phenyl-1*H*-pyrazole and bpy = 2, 2′-bipyridine), 2, 2′-bipyridinebis(1-phenyl-1*H*-pyrazole)iridium(III) hexafluorophosphate Reported [47]

**4: [Ir(pbt)2(cphen)](PF6)** (where pbt = 2-phenylbenzo[d]thiazole and cphen = 5-chloro-1, 10-phenanthroline), 5-chloro-1, 10-phenanthrolinebis(2-phenylbenzo[d]thiazole)iridium(III) hexafluorophosphate Yield: 81.0%. ^1^H NMR (400 MHz, DMSO-*d*_6_) δ 9.07 (d, *J* = 8.4 Hz, 1H), 8.91 (d, *J* = 8.0 Hz, 1H), 8.72 (s, 1H), 8.52 (d, *J* = 1.2 Hz, 1H), 8.43 (d, *J* = 1.2 Hz, 1H), 8.27 (q, *J* = 5.2 Hz, 1H), 8.20–8.15 (m, 3H), 8.07 (d, *J* = 7.6 Hz, 2H), 7.33 (t, *J* = 8.8 Hz, 2H), 7.21–7.19 (m, 2H), 7.02–6.95 (m, 4H), 6.39 (t, *J* = 6.4 Hz, 2H), 5.76 (d, *J* = 8.4 Hz, 2H); ^13^C NMR (100 MHz, DMSO-*d*_6_) 181.3, 181.2, 152.1, 151.6, 149.5, 149.2, 148.5, 147.5, 146.0, 140.1, 140.0, 138.8, 136.3, 132.8, 132.7, 132.2, 132.1, 130.9, 130.0, 128.7, 128.4, 128.1, 128.0, 127.9, 127.6, 127.1, 125.9, 124.6, 123.3; MALDI-TOF-HRMS: Calcd. for C_38_H_23_ClIrN_4_S_2_[M–PF_6_]^+^: 827.0681, found: 827.2160; Anal.: (C_38_H_23_ClIrN_4_S_2_PF_6_) C, H, N: calcd. 46.94, 2.38, 5.76; found. 46.76, 2.26, 5.76.

**5: [Ir(pq)2(pzphen)](PF6)** (where pq = 2-phenylquinoline and pzphen = pyrazino[2, 3-f][1, 10]phenanthroline), pyrazino[2, 3-f][1, 10]phenanthrolinebis(2-phenylquinoline)iridium(III) hexafluorophosphate Reported [32]

**6: [Ir(dfppy)2(dcdppz)](PF6)** (where dfppy = 2-(2, 4-difluorophenyl)pyridine and dcdppz = 11, 12-dichlorodipyrido[3, 2-a:2′,3′-c]phenazine), 11, 12-dichlorodipyrido[3, 2-a:2′,3′-c]phenazinebis(2-(2, 4-difluorophenyl)pyridine)iridium(III) hexafluorophosphate Yield: 78.5%. ^1^H NMR (400 MHz, Acetone-*d*_6_) δ 9.85 (d, *J* = 7.2 Hz, 2H), 8.72–8.68 (m, 4H), 8.41 (d, *J* = 8.4 Hz, 2H), 8.30–8.27 (m, 2H), 8.03 (t, *J* = 8.0 Hz, 2H), 7.95 (t, *J* = 6.0 Hz, 2H), 7.12–7.09 (m, 2H), 6.85–6.80 (m, 2H), 5.91 (d *J* = 8.4 Hz, 2H); ^13^C NMR (100 MHz, Acetone-*d*_6_) 165.8, 165.7, 164.8, 164.7, 163.6, 163.3, 163.2, 161.1, 154.5, 154.4, 154.3, 151.2, 151.0, 142.3, 142.0 140.8, 137.3, 137.0, 132.0, 131.2, 129.8, 125.0, 124.7, 124.5, 115.0, 114.8, 100.2, 99.9, 99.6; MALDI-TOF-HRMS: Calcd. for C_40_H_20_Cl_2_F_4_IrN_6_[M–PF_6_]^+^: 923.0692, found: 923.0699; Anal.: (C_40_H_20_Cl_2_F_4_IrN_6_PF_6_) C, H, N: calcd. 44.95, 1.89, 7.86; found. 44.94, 1.88, 7.68.

### Protein expression and fluorescence anisotropy assay

Expression of *h*DM2_17–126_ recombinant fragment was performed as described previously [33]. Fluorescence anisotropy assays were used to study the displacement of p53 from *h*DM2 by organometallic compounds. The experiments were performed in triplicate in a volume of 60 μL of the assay buffer (40 mM Na_3_PO_4_, pH 7.50, 200 mM NaCl, 0.02 mg/mL BSA) in black flat-bottom 384-well plates. Different concentrations of organometallic compounds were diluted in the assay buffer containing 150 nM *h*DM2 and 50 nM p53_15–31Flu_ (Ac-SQETFSDLWKLLPENNVC(Flu)-NH_2_, Peptide Protein Research Ltd.) and incubated at room temperature for 30 min. The intensity and anisotropy was monitored at 480ex/535em (5 nM bandwidth) using a Perkin Elmer EnvisionTM 2103 MultiLabel plate reader. The data was analyzed according to published methods [48, 49].

### NanoBRET protein:protein interaction assay

A NanoBRET protein:protein interaction system (Promega) was used to detect p53/*h*DM2 protein-protein interactions by measuring energy transfer from a bioluminescent protein donor to a fluorescent protein acceptor. A375 cells were incubated with a transfection mixture of 2 μg of p53-HaloTag fusion gene and 0.2 μg of NanoLuc-*h*DM2 fusion gene in Opti-MEM^®^ I reduced serum medium with TurboFect transfection reagent (Thermo Scientific) in 6-well plates for 20 h at 37°C, 5% CO_2_. The transfected cells were replated in a white 96-well plate with HaloTag^®^ NanoBRET 618 ligand (100 nM) in Opti-MEM^®^ I reduced serum medium with 4% fetal bovine serum (FBS) and incubated overnight. **1** was the added directly to the culture medium at different concentrations and the plate was incubated for further 6 h. 25 μL of 1X Nano-Glo^®^ substrate in Opti-MEM^®^ I reduced serum medium was added to the wells. The plate was then measured using 460 nm filter and 610 nm filters in SpectraMax M5 microplate reader (Molecular Devices).

### Pull-down assay

A375 cells seeded in 6-well plates were co-transfected with pcDNA-Flag-p53 (Addgene) and pCMV-myc3-*h*DM2 (Addgene) for 6 h using TurboFect transfection reagent following the instruction from manufacturer. Cells were then cultured in Dulbecco's Modified Eagle's Medium (DMEM) complete medium overnight. Serial dilutions of organometallic compounds in low FBS buffer were placed into the wells, and incubated with cells for additional 6 h. Protein samples were collected, and the concentration in the supernatant was determined with the bicinchoninic acid (BCA) method. The organometallic compounds of p53/*h*DM2 in the protein samples were pulled down using anti-Flag magnetic beads to capture the FLAG fusion proteins (Sigma) as previously described [50, 51]. The protein-binding beads were washed three time with TBS buffer (50 mM Tris HCl, 150 mM NaCl, pH 7.4) to remove non-specifically bound proteins, and subsequently subjected to the sodium dodecyl sulfate-polyacrylamide gel electrophoresis (SDS-PAGE) by detection with anti-Flag (1:1000, Sigma-Aldrich) and anti-myc antibodies (1:1000, Beyotime).

### Immunoblotting

A375 cells seeded in 6-well plates were treated with vehicle, NVP-CGM097 or different concentrations of **1** for 6 h. Cells were then harvested and the quantity of each protein samples was measured by the BCA method. Same aliquot (30 μg) of each protein sample was separated by SDS-PAGE according to the reported procedure of immunoblotting [50]. The signals were visualized using Enhanced chemiluminescent Plus reagents (GE Healthcare) and analyzed by Image Lab. The primary antibodies used were: anti-PUMA (1:500, Santa Cruz), anti-GADD45α (1:500, Santa Cruz), anti-p21 (1:500, Santa Cruz), anti-GAPDH (1:500, Santa Cruz), anti-p53 (1:500, Santa Cruz) and anti-*h*DM2 antibodies (1:500, Santa Cruz).

### Dual luciferase reporter assay

A375 cells were co-transfected with p53 luciferase reporter gene (Addgene) and *Renilla* luciferase (*Rluc*) control reporter vectors at the ratio of 10:1 for 6 h in a 6-well plate. After transfection, cells were cultivated for another 18 h in the complete DMEM medium. Cells were then plated in a 96-well plate overnight. Serial dilutions of **1** were then added to the wells and the plate was incubated for 6 h. Luciferase reporter activity was then measured according to instruction of a dual reporter assay system (Progema) as previous reported [50].

### TUNEL assay

The TUNEL assay was performed to measure apoptosis in cells induced by the treatment of **1**. Briefly, A375 cells were incubated with **1** for 6 h. Apoptosis in cells was measured using the APO-BrdU^™^ TUNEL assay kit (Thermo Fisher) according to the manufacturer's instruction. After fixing cells by the addition of 100 μL of 1% (w/v) paraformaldehyde in phosphate-buffered saline (PBS) and permeabilization with 70% (v/v) ice-cold ethanol, the cells were plated on ice for 30 min. 50 μL of DNA-labeling solution (10 μL of reaction buffer, 0.75 μL of TdT enzyme, 8.0 μL of BrdUTP and 31.25 μL of dH_2_O) was added to wells. The plate was incubated with the DNA-labeling solution for 1 h at 37°C with shaking every 15 min. Then, the cells were washed with 10 μL of rinse buffer three times before staining with diluted Fluor^®^ 488 dye-labeled anti-BrdU antibody solution (1:20) for 30 min at room temperature. 100 μL of propidium iodide/RNase A staining buffer was then added to wells and the cells were incubated for an additional 30 min. The samples were analyzed using a GE Healthcare Life Sciences IN Cell Analyzer 2000.

### Caspase-Glo^®^ 3/7 assay

The activities of caspase 3/7 were detected using Caspase-Glo^®^ 3/7 assay kit (Promega). Following 6 h treatment of different concentrations of **1**, a 96-well luminometer plate containing A357 cells was removed from the incubator and equilibrated to room temperature for 30 min. 100 μL of Caspase-Glo^®^ 3/7 reagent was added to each well and the plate was shaking for 30 sec at room temperature. The luminescence signaling of each sample was collected in SpectraMax M5 microplate reader.

### Cell proliferation assay

Inhibition of cell growth was measured using the MTT reagent (3-(4, 5-dimethylthiazol-2-yl)-2, 5-diphenyltetrazolium bromide). Different cell lines with variable expression levels of p53 protein (A375, A549, HeLa, A2780, A431, MCF7, MD-MBA-231, MD-MBA-468, T47D and H1299 cell lines) were plated onto 96-well plates at a density of 4, 000 cells/well overnight prior to the addition of **1** at different concentrations ranging from 0.01–100 μM for a further 72 h. 10 μL MTT reagent (5 mg/mL) was added to the wells and the cells were incubated for an additional 4 h before 100 μL of DMSO was added to the wells. The absorbance of each well was read on a SpectraMax M5 microplate reader (Molecular Devices) at the wavelength of 570 nm.

## SUPPLEMENTARY MATERIALS FIGURES AND TABLES


