# Supplementary material for: Inhibition of the p53/hDM2 protein-protein interaction by cyclometallated iridium(III) compounds
Source: Oncotarget. 2016 Feb 13;7(12):13965–75. doi: 10.18632/oncotarget.7369 (PMC4924691; doi:10.18632/oncotarget.7369)
Supplement: Supplementary file 1 [file oncotarget-07-13965-s001.pdf]

Inhibition of the p53/*h*DM2 protein-protein interaction by cyclometallated iridium(III) compounds

Supplementary Materials

Synthesis of cyclometallated iridium (III) compounds

Preparation of the C^N ligand of 1 and 2

A solution of 1-bromo-4-ethylbenzene (0.37 g, 2.0 mmol) in 10 mL toluene was added bis(pinacolato) diboron (0.61 g, 2.4 mmol) and sodium acetate (0.25 g, 3.0 mmol), followed by the addition of catalytic amounts of Pd(dppf)Cl_2 under nitrogen atmosphere. The resulting mixture was allowed to heat 100°C for 5 hours. The reaction was monitored by Thin Layer Chromatography (TLC) (PE : EA = 8:1). Subsequently the reaction mixture was cooled to room temperature, diluted with EtOAc (40 mL), and filtered to remove the solid. The filtrate was washed with H_2O (2 × 15 mL) and brine (2 × 15 mL). The organic layer was dried by anhydrous Na_2SO_4, and the solvent was removed in vacuum to get the crude product which was purified by silica gel column (PE : EA = 20:1~8:1) to yield 8 (0.42 g, yield: 86.2%) as a colorless oil. ^{1}H NMR (400 MHz, $CDCl_3$) δ 7.75 (d, *J* = 8.0 Hz, 2H), 7.22 (d, *J* = 8.0 Hz, 2H), 2.67 (q, *J* = 7.6 Hz, 2H), 1.35 (s, 12 H), 1.24 (t, *J* = 8.0 Hz, 3H); ^{13}C NMR (100 MHz, $CDCl_3$) δ 147.8, 134.9, 127.4, 83.6, 29.1, 24.9, 15.5.

A solution of 8 (0.40 g, 1.7 mmol) in 15 mL ethanol was added 2-bromopyridine (0.27 g, 1.7 mmol) and potassium carbonate (0.36 g, 2.6 mmol), followed by the addition of catalytic amounts of $Pd(PPh_3)_4$ under nitrogen atmosphere. The resulting mixture was allowed to heat to reflux for overnight. The reaction was monitored by TLC (PE : EA = 5:1). After the reaction, the mixture was cooled to room temperature, diluted with EtOAc (50 mL), and filtered to remove the solid. The filtrate was washed with H_2O (2 × 15 ml) and brine (2 × 15 ml). The organic layer was dried by anhydrous Na_2SO_4, and the solvent was removed in vacuum to get the crude product which was purified by silica gel column (PE : EA = 10:1~5:1) to yield 9 (0.29 g, yield: 93.2%) as a colorless oil. ^{1}H NMR (400 MHz, $CDCl_3$) δ 8.67–8.66 (m, 1H), 7.91 (d, *J* = 6.4 Hz, 2H), 7.75–7.69 (m, 2H), 7.31 (d, *J* = 8.0 Hz, 2H), 7.21–7.18 (m, 1H), 2.70 (q, *J* = 7.6 Hz, 2H), 1.28 (t, *J* = 7.6 Hz, 3H); ^{13}C NMR (100 MHz, $CDCl_3$) δ 157.5, 149.6, 145.3, 136.9, 136.7, 128.3, 126.9, 121.8, 120.3, 28.9, 15.5. HRMS: Calcd. for $C_{13}H_{13}N[M+H]^+$: 184.1126 Found: 184.1129.

Preparation of the N^N ligands of compounds 5 and 6

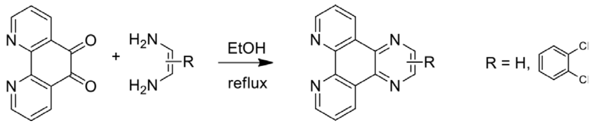

A mixture of 1, 10-phenanthroline-5, 6-dione (5.0 mmol) and diamine (6.0 mmol, 1.2 eq.) in ethanol were heated to reflux for overnight. After the reaction mixture was slowly cooled down to room temperature, the solid that precipitated from the solution were filtered off, washed with cold ethanol, and suction dried to obtain the desired products in pure form with yields of 86 and 92% directly used in next step.

Preparation of the dimer compounds $[(C\^N)_2Ir(\mu\text{-}Cl)]_2$

A mixture of iridium(III) chloride (150 mg, 0.43 mmol) and corresponding C^N ligands (0.95 mmol, 2.2 eq.) in methoxyethanol: deionized H_2O (3:1, 12 mL) was allowed to heat to reflux overnight under nitrogen protection. The mixture was cooled to ambient temperature after the reaction. And the formed solid was collected by filtration, washed with deionized H_2O (2 × 50 mL) and diethyl ether (2 × 30 mL) and dried to afford the corresponding dimer compounds.

Preparation of the final cyclometallated iridium(III) compounds

A suspension of $[(C\^N)_2Ir(\mu\text{-}Cl)]_2$ (0.2 mmol) and corresponding N^N ligands (0.24 mmol, 2.2 eq.) in DCM:MeOH (1:1, 16 mL) was heated to reflux for 6 h. The resulting solution was cooled to ambient temperature, and filtered to remove unreacted cyclometallated dimer. Afterwards an aqueous solution of ammonium hexafluorophosphate was added to the filtrate, and the solvent was removed in vacuum until the solid occurred. The precipiate was collected and washed with deionized H_2O (2 × 50 mL) followed by diethyl ether (2 × 30 mL). The mixture was purified by silica gel column dichloromethane as the eluent to afford the titled compounds.

Stability analysis

1 was stored in $[d_6]$DMSO/D_2O (v/v = 9:1) at 298K for seven days, and was determined by ^1H NMR spectroscopy. ^1H NMR experiments were carried out on a 400 MHz (^1H) Bruker instrument. Additionally, **1** was also stored in acetonitrile/Tris-HCl buffer (v/v = 8:2, 20 μM) 298K for seven days. Absorption spectra were recorded on Cary UV-100 Spectrophotometer.

Photophysical measurement

The photophysical properties including UV absorbance, emission spectra and lifetime of iridium(III)-cyclometallated compounds were performed on a PTI TimeMaster C720 Spectrometer (Nitrogen laser: pulse output 337 nm) based on the reported methods [1].

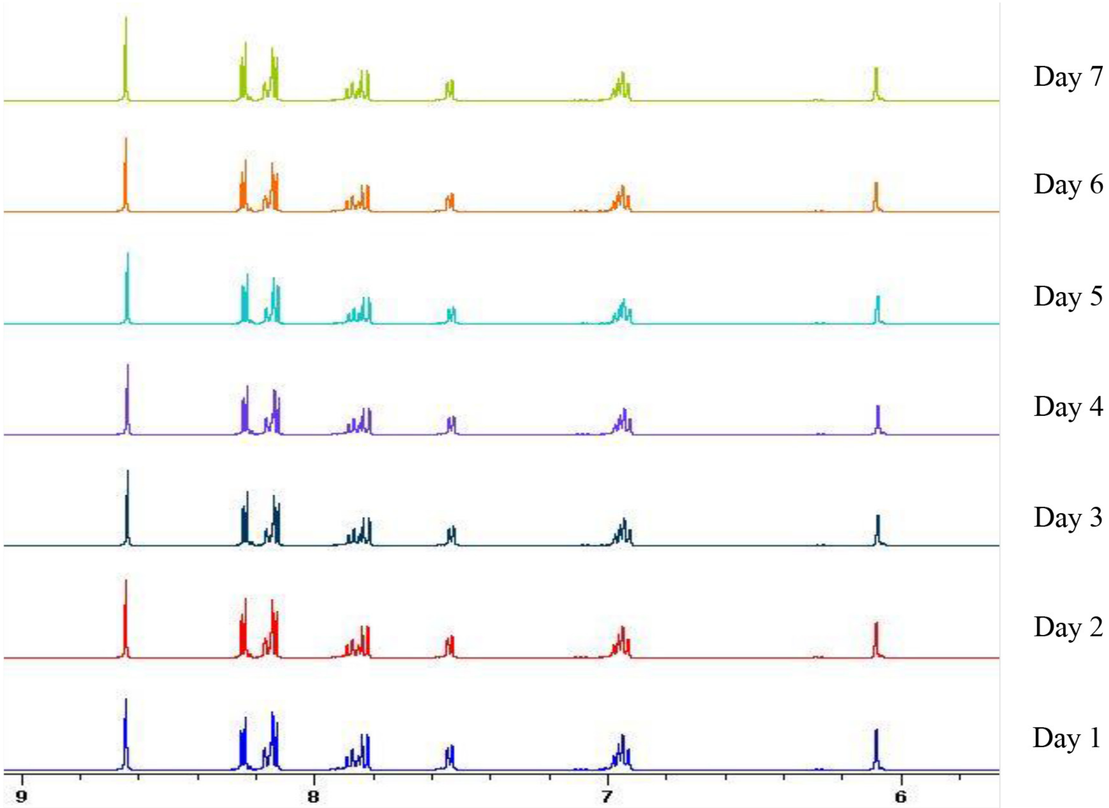

Supplementary Figure S1: Aromatic region of the ^1H NMR spectra of 1 at a concentration of 5 mM in 90% $[d_6]$ DMSO/10% D_2O at 298 K over 7 days.

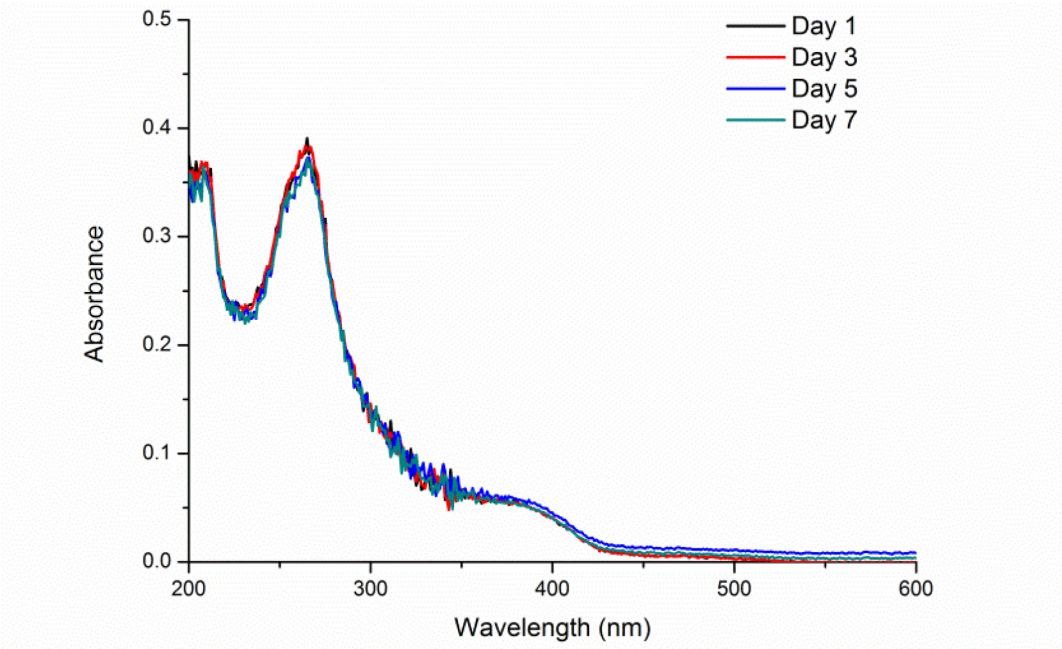

Supplementary Figure S2: UV/Vis absorption of 1 at a concentration of 20 µM in 80% acetonitrile/20% 20 mM Tris-HCl buffer (containing 20 mM NaCl, pH 7.5) at 298 K over 7 days.

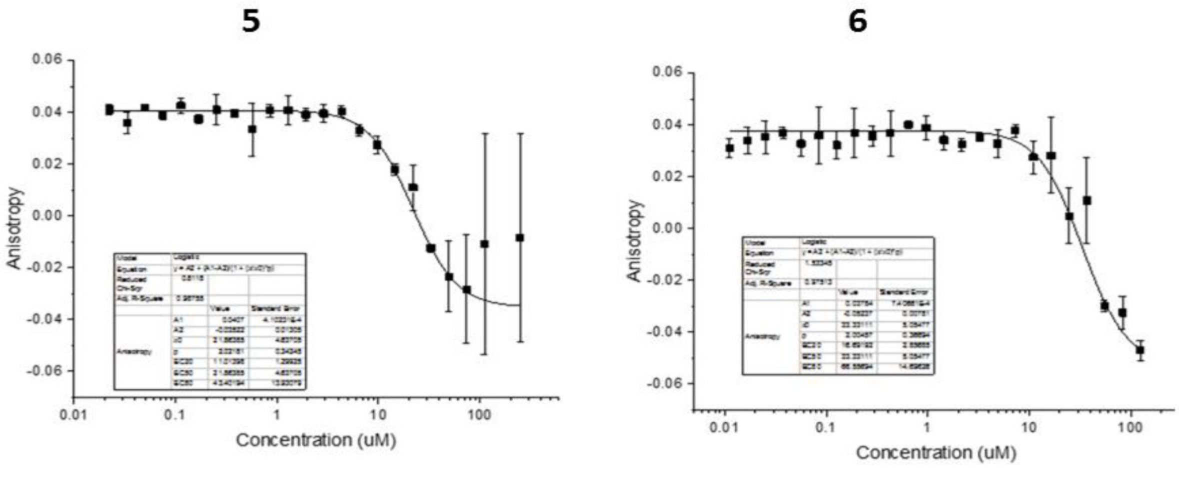

Supplementary Figure S3: FA titration data of 5 and 6. 5 and **6** was incubated together with 150 nM *h*DM2$_{17–126}$ recombinant fragment and 50 nM fluorescein-labelled p53 peptide (p53$_{15–31Flu}$), the fluorescence anisotropy was measured at 480ex/535em.

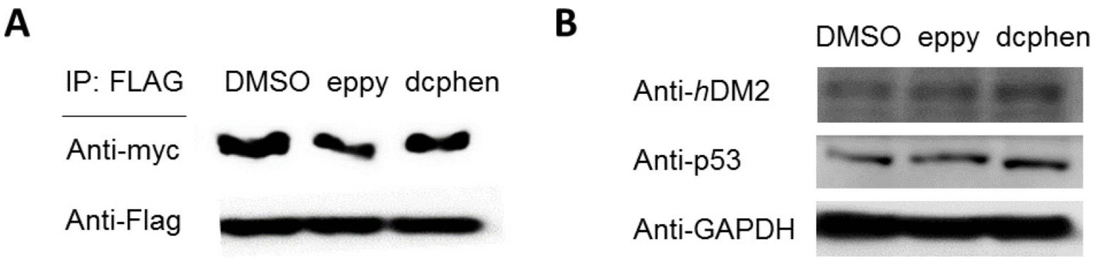

Supplementary Figure S4: (**A**) Ligand eppy and dcphen have no effect on the interaction of p53/*h*DM2 in A375 cells. **(B)** Ligand eppy and dcphen have no effect on the protein expression levels of p53 and *h*DM2 in A375 cells.

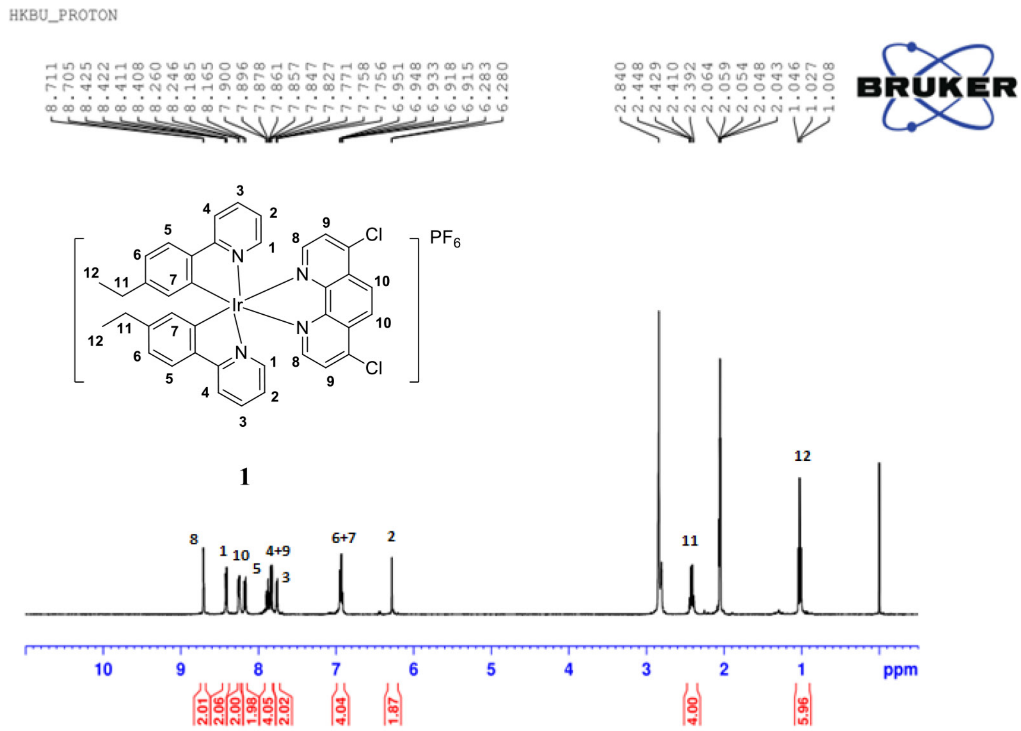

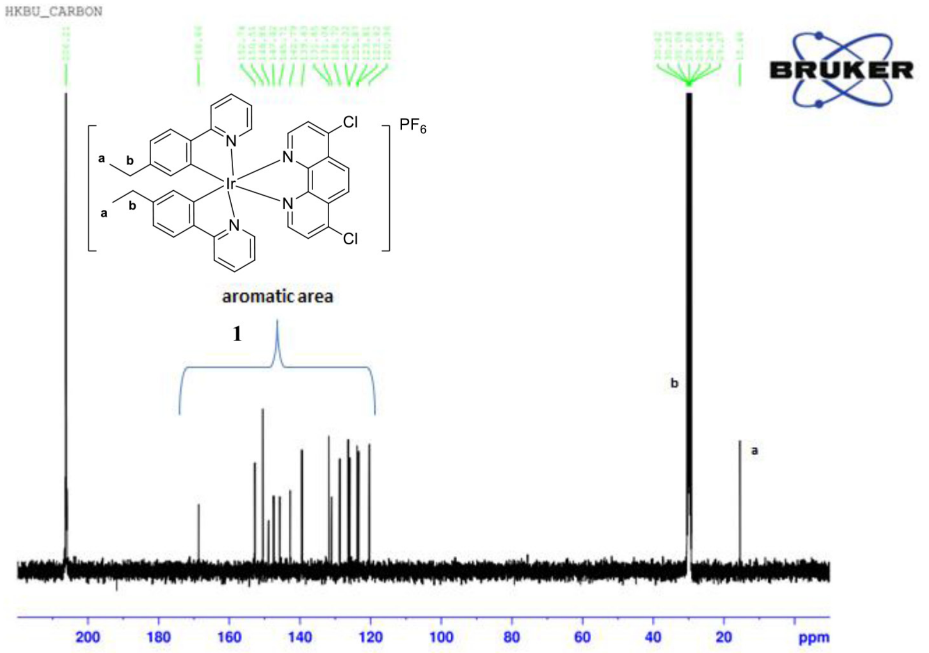

Supplementary Figure S5: NMR signal assignment of 1.

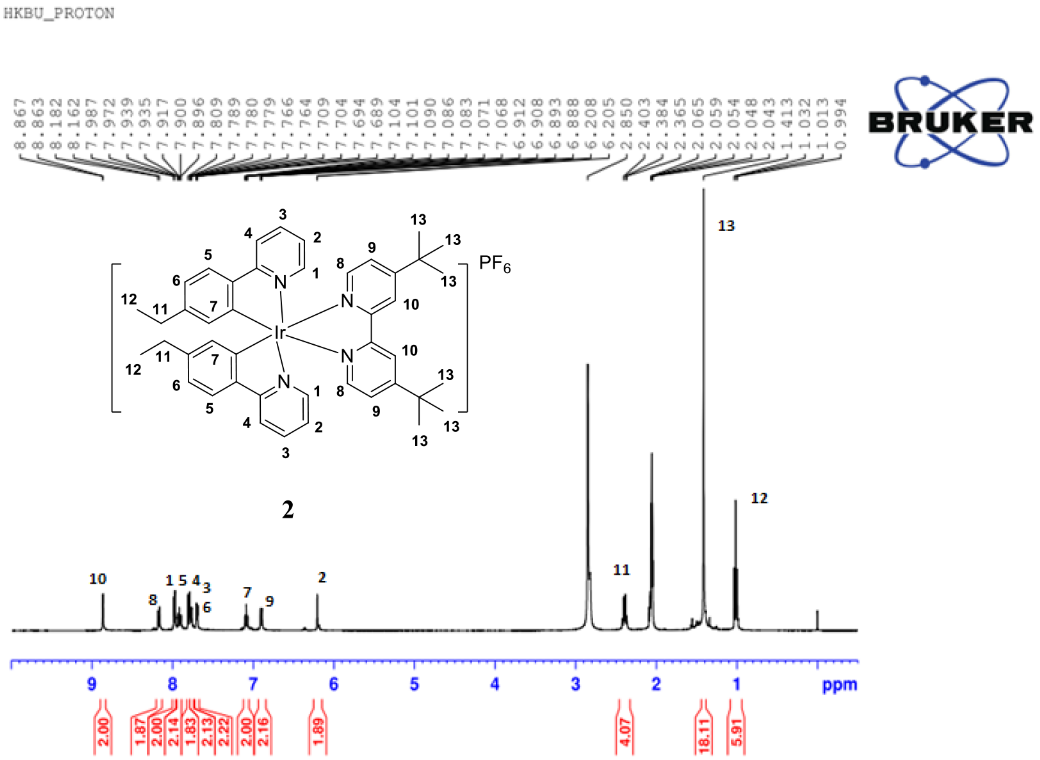

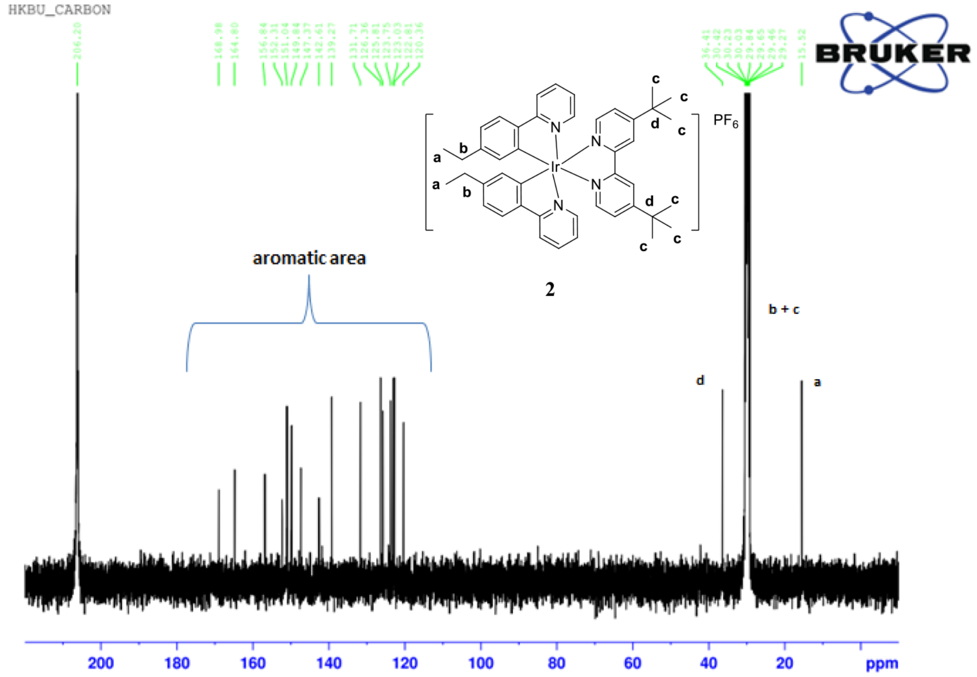

Supplementary Figure S6: NMR signal assignment of 2.

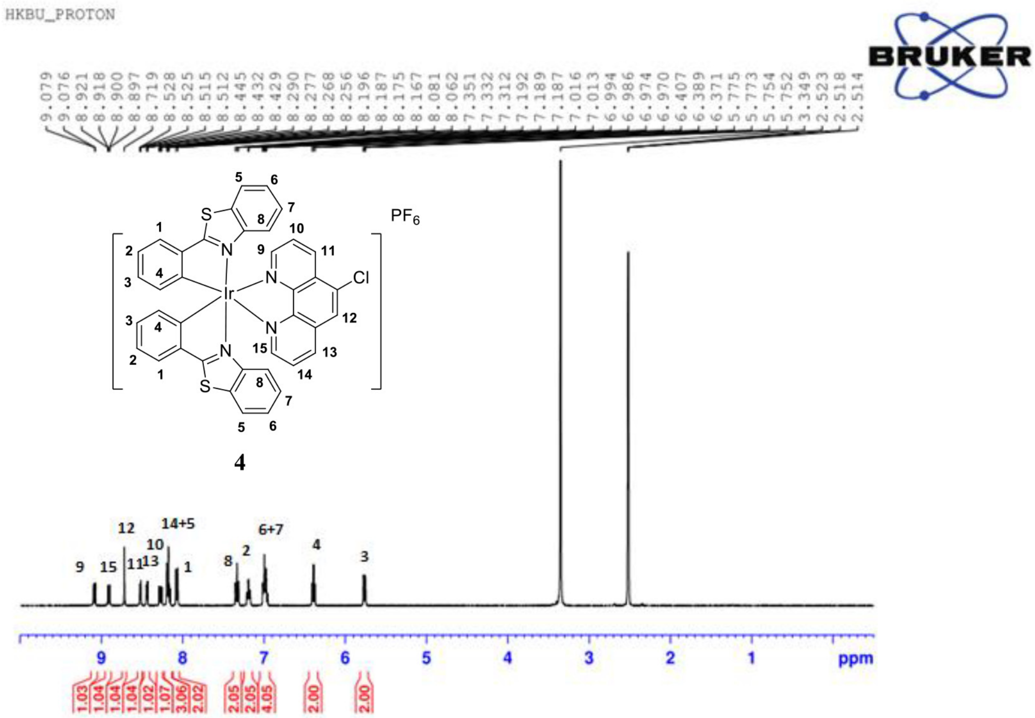

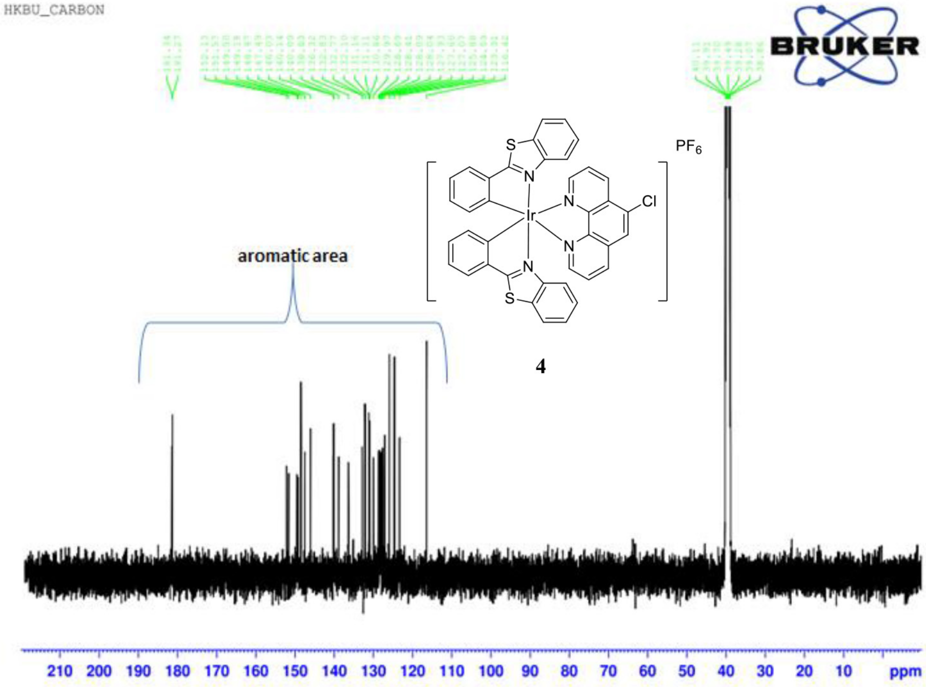

Supplementary Figure S7: NMR signal assignment of 4.

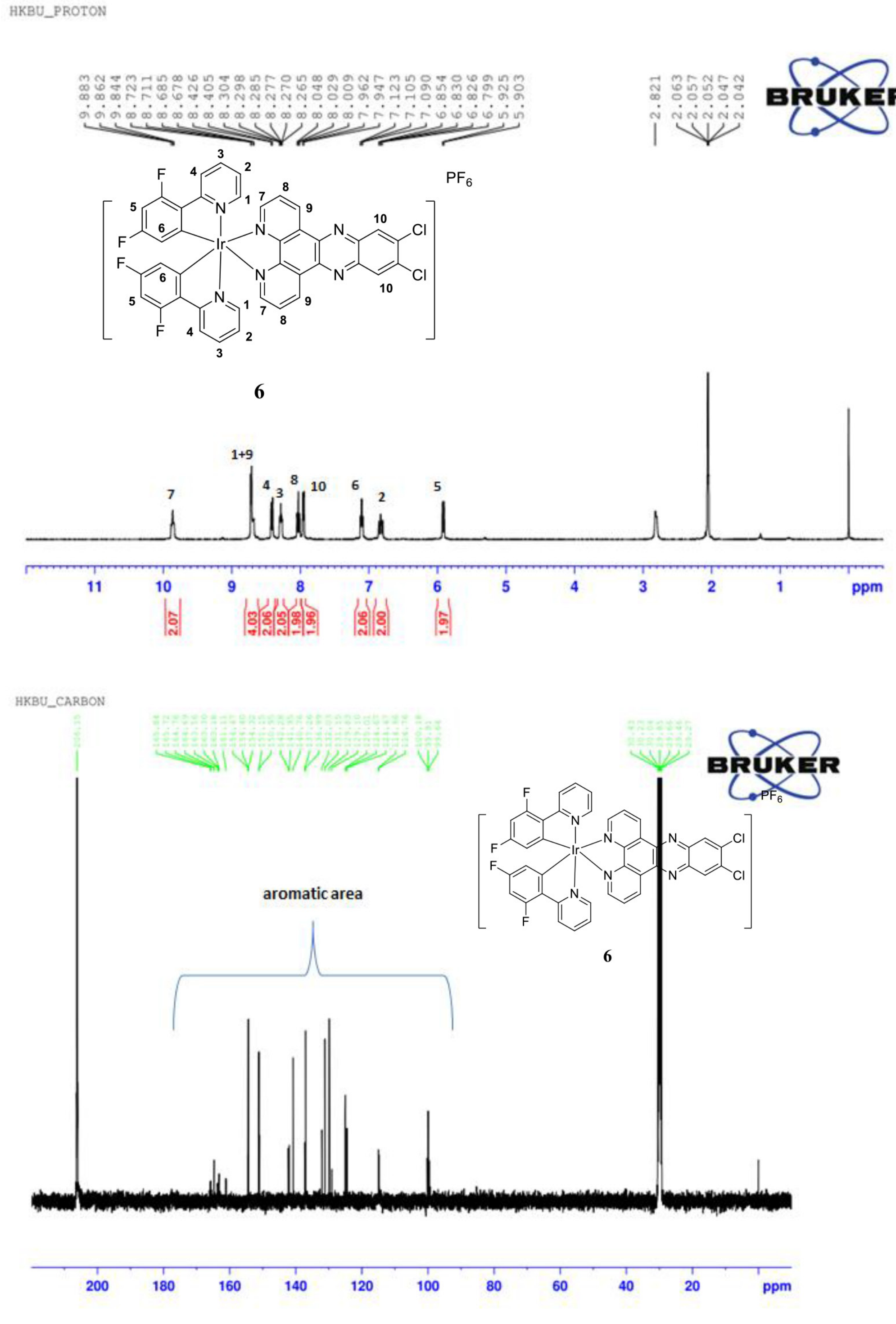

Supplementary Figure S8: NMR signal assignment of 6.

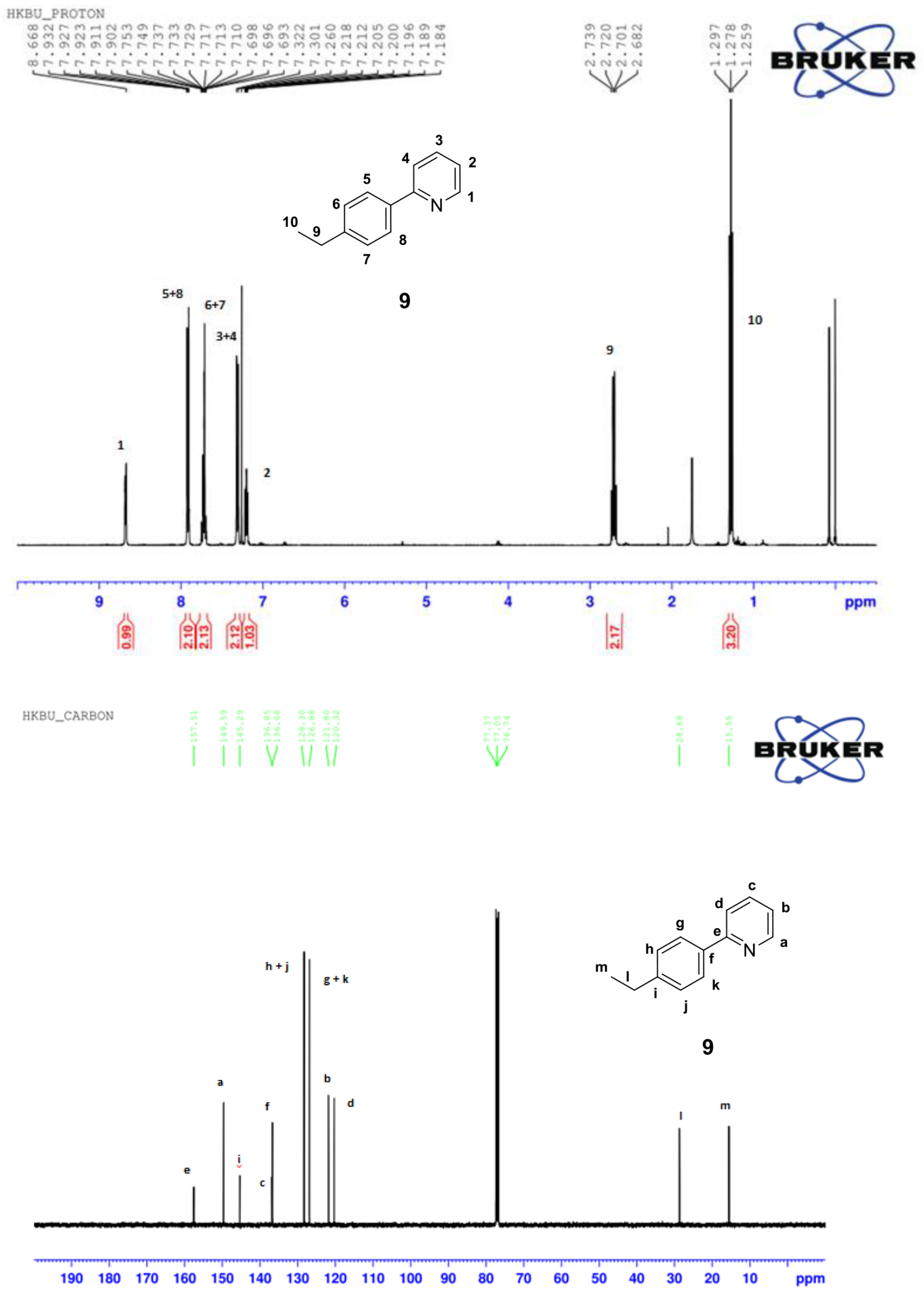

Supplementary Figure S9: NMR signal assignment of 9.

Supplementary Table S1: Photophysical properties of cyclometallated iridium(III) compounds 1–6

Cyclometallated iridium(III) compounds	Quantum yield	λ_{em}/ nm	Lifetime/μs	UV/vis absorption λ_{abs}/nm (ε/dm^3·mol^{-1}·cm^{-1})
**1**	0.063	639	$4.577 \pm 5.090 \times 10^{-3}$	267 (7.95×10^3)
**2**	0.181	585	$4.508 \pm 5.638 \times 10^{-3}$	209 (8.20×10^3), 257 (1.15×10^4), 285 (1.43×10^4), 299 (6.35×10^3)
**3**	0.275	574	$4.399 \pm 3.394 \times 10^{-3}$	246 (1.07×10^4)
**4**	0.142	608	$3.459 \pm 1.297 \times 10^{-2}$	203 (1.50×10^4), 229 (1.23×10^4), 270 (8.10×10^3),
**5**	0.067	568	$4.610 \pm 7.413 \times 10^{-3}$	262 (3.79×10^4), 279 (2.88×10^4), 334 (1.13×10^4)
**6**	0.010	554	$3.856 \pm 6.450 \times 10^{-3}$	213 (1.03×10^4), 252 (1.11×10^4), 275 (1.65×10^4), 316 (5.10×10^3),

Supplementary Table S2: The toxicity of 1 on different human cancer cell lines as determined by an MTT assay

Cell lines	p53 status	IC_{50} values of 1 (μM)
Human malignant melanoma		
A375	wt	0.6468 ± 0.03541
Human colon carcinoma		
A431	wt	0.4164 ± 0.03742
Human breast carcinoma		
MCF7	wt	0.5469 ± 0.04096
MD-MBA-231	mut	0.8348 ± 0.04286
MD-MBA-468	mut	1.888 ± 0.02335
T47D	mut	0.7832 ± 0.4826
Human lung carcinoma		
A549	wt	0.2320 ± 0.02183
H1299	null	2.204 ± 0.03826
Human ovarian carcinoma		
A2780	wt	0.4950 ± 0.03202
Human cervix carcinoma		
HeLa	wt	0.4799 ± 0.04474

REFERENCES

1. Lin S, Gao W, Tian ZR, Yang C, Lu LH, Mergny JL, Leung CH, Ma DL. Luminescence switch-on detection of protein tyrosine kinase-7 using a G-quadruplex-selective probe. Chem Sci. 2015; 6:4284–4290.
